# Transplantation of neural stem cells improves recovery of stroke-affected mice and induces cell-specific changes in GSDMD and MLKL expression

**DOI:** 10.3389/fnmol.2024.1439994

**Published:** 2024-08-15

**Authors:** Damir Lisjak, Ivan Alić, Iva Šimunić, Dinko Mitrečić

**Affiliations:** ^1^Laboratory for Stem Cells, Department for Regenerative Neuroscience, Croatian Institute for Brain Research, School of Medicine, University of Zagreb, Zagreb, Croatia; ^2^Department of Anatomy, Histology and Embryology, Faculty of Veterinary Medicine, University of Zagreb, Zagreb, Croatia

**Keywords:** stroke, neural stem cells, cell transplantation, MRI, pyroptosis, necroptosis, neuroinflammation

## Abstract

**Introduction:**

Stroke, the second leading cause of death and disability in Europe, is primarily caused by interrupted blood supply, leading to ischemia–reperfusion (IR) injury and subsequent neuronal death. Current treatment options are limited, highlighting the need for novel therapies. Neural stem cells (NSCs) have shown promise in treating various neurological disorders, including stroke. However, the underlying mechanisms of NSC-mediated recovery remain unclear.

**Methods:**

Eighty C57Bl/6–Tyrc-Brd mice underwent ischemic stroke induction and were divided into four groups: sham, stroke-affected, stroke-affected with basal cell medium injection, and stroke-affected with NSCs transplantation. NSCs, isolated from mouse embryos, were stereotaxically transplanted into the stroke-affected brains. Magnetic resonance imaging (MRI) and neurological scoring were used to assess recovery. Immunohistochemical analysis and gene expression assays were performed to evaluate pyroptosis and necroptosis markers.

**Results:**

NSC transplantation significantly improved neurological recovery compared to control groups. In addition, although not statistically significant, NSCs reduced stroke volume. Immunohistochemical analysis revealed upregulation of Gasdermin D (GSDMD) expression post-stroke, predominantly in microglia and astrocytes. However, NSC transplantation led to a reduction in GSDMD signal intensity in astrocytes, suggesting an effect of NSCs on GSDMD activity. Furthermore, NSCs downregulated Mixed Lineage Kinase Domain-Like Protein (*Mlkl*) expression, indicating a reduction in necroptosis. Immunohistochemistry demonstrated decreased phosphorylated MLKL (pMLKL) signal intensity in neurons while stayed the same in astrocytes following NSC transplantation, along with increased distribution in microglia.

**Discussion:**

NSC transplantation holds therapeutic potential in stroke recovery by targeting pyroptosis and necroptosis pathways. These findings shed light on the mechanisms underlying NSC-mediated neuroprotection and support their further exploration as a promising therapy for stroke patients.

## 1 Introduction

Stroke is the second leading cause of death and long-term disability in Europe (Naghavi et al., 2015; Webb et al., 2021). In over 80% of cases, it is caused by a decreased or interrupted blood supply. The pathophysiological elements which make up a complex chain of detrimental effects can be described as an ischemia–reperfusion (IR) injury, which leads to an almost immediate death of some neurons and glia cells, combined with prolonged inflammation. The extent of damage correlates to subsequent neurological dysfunction (Thom et al., 2006). The current treatment options, unfortunately applicable only in 5% of cases are limited to mechanical thrombectomy and intravenous thrombolysis by tissue plasminogen activator (tPA) (Leng and Xiong, 2019; Jang et al., 2021).

Since all described treatment options of ischemic stroke are reperfusion options and are available for only a specific time after the stroke since IR injury later causes more damage than use, it has become a popular field of research. Hypoxia causes significant changes in cellular metabolism because of the limited ATP synthesis due to mitochondrial dysfunction, leading to disruptions in ion concentrations of Na^+^, K^+^, and Ca^2+^. Furthermore, it modifies pathways such as xanthine oxidase, hypoxia-inducible factor 1α, NADPH, NAD+, nitric oxide synthase (NOS) that in the end increase production of reactive oxygen species (ROS) (Wu et al., 2018). Hypoxia, HIF1α, ROS, and mitochondrial dysfunction trigger pro-inflammatory cytokines (IL-1, IL-6, TNF), caspases, and activate the NF-κB pathway, leading to apoptosis, necroptosis, pyroptosis, and autophagy. Cell death releases damage-associated molecular patterns (DAMPs), ROS, and NF-κB pathway activation, which are key in activating microglia, astrocytes, and leukocytes, further driving the inflammatory response (Jurcau and Simion, 2021).

Microglia adopt different phenotypes, M1 pro-inflammatory and M2 anti-inflammatory, to respond to the damage. Following transient middle cerebral artery occlusion (MCAO) in mice, microglial infiltration peaks at 48–72 h, where they migrate to the ischemic lesion and cluster near neurons, aiding in the removal of damaged cells (Jayaraj et al., 2019). In the chronic phase, M2 microglia enhance neuroplasticity and promote neurogenesis by secreting neurotrophic factors (Singhal and Baune, 2017).

Neural stem cells (NSCs) represent a natural choice for treatment of brain diseases and, since their first application, measurable beneficial effects keep being reported, including but not limited to Parkinson’s disease (Redmond et al., 2007), Lysosomal Storage Diseases (De Filippis and Delia, 1982), Amyotrophic Lateral Sclerosis (Mitrečić et al., 2010), and Multiple Sclerosis (Brown et al., 2021). NSCs display an inherent mechanism for rescuing dysfunctional neurons (Ourednik et al., 2002) which is, at least partly, achieved through the secretion of growth factors, such as brain-derived neurotrophic factor (BDNF), nerve growth factor (NGF) and vascular endothelial growth factor (VEGF) (Nicaise et al., 2011; Červenka et al., 2021). Several studies reported successful transplantation (stereotaxic or intravascular) of NSCs into the animal models of stroke and, a great majority of them, reported improved recovery of the treated animals (Shen et al., 2010; Dai et al., 2013; Mine et al., 2013; Huang et al., 2014; Kondori et al., 2020). However, some of them showed significant improvements to certain elements of pathophysiological processes, while a majority of them reported a reduction in inflammation (Bacigaluppi et al., 2009; Shen et al., 2010; Mine et al., 2013; Huang et al., 2014; Hamblin et al., 2022). Only a few studies reported the reduction of apoptosis, either as a mechanism of improving condition of the host (Shen et al., 2010; Xue et al., 2019), or as a mechanism linked to improved survival of transplant within the tissue affected by ischemia (Kosi et al., 2018). Interestingly, some studies found that the improvements in the health status positively correlated with the decrease in stroke volume (Bacigaluppi et al., 2009; Huang et al., 2014; Kondori et al., 2020), while others found no such effects (Cheng et al., 2015). At the same time, while significant improvements following transplantation of human NSCs to patients have also been reported in clinical trials (Kalladka et al., 2016; Steinberg et al., 2016; Muir et al., 2020), the mechanism by which stem cells achieve these positive effects still remains elusive.

Pyroptosis is a caspase-1 mediated type of cell death characteried by DNA cleavage, actin cytoskeleton destruction and rupture of plasma membrane resulting in the release of proinflammatory cellular content (Bergsbaken et al., 2009). The main effector is Gasdermin D (GSDMD) which, when cleaved, inserts itself into the plasma membrane where it forms oligomeric pores and, subsequently onsets the process of osmotic lysis (Shi et al., 2015). Necroptosis is programmed necrosis mediated by several mediators, including death receptors, members of the tumor necrosis factor receptor superfamily (Dhuriya and Sharma, 2018). Similar to pyroptosis, it also causes the loss of cell membrane integrity and induces the release of cell content causing local inflammation (Liu et al., 2018). The main effector of necroptosis is phosphorylated mixed lineage kinase domain-like protein (pMLKL) which, when translocated to the plasma membrane causes significant membrane permeabilization (Flores-Romero et al., 2020).

The goal of this research is to clarify the role of pyroptosis and necroptosis, through the key factors of these processes, GSDMD and pMLKL, in the pathophysiology of stroke and explore the approach in which NSC transplantation affects these processes. Moreover, the investigation of GSDMD and pMLKL expression and localization following NSC transplantation in stroke-affected mice aims to provide new insights into the mechanisms by which NSCs achieve their neuroprotective advantages and prospective treatment approaches.

## 2 Materials and methods

### 2.1 Experimental animals and stroke induction

In total, 82 animals of a C57Bl/6–Tyr^c-Brd^ mouse strain (The Jackson Laboratory, Bar Harbor, ME, USA) were used, including 80 males equally divided into 4 groups (A - sham, B - stroke affected animals, C - stroke affected animals injected with basal cell medium and D - stroke affected animals injected with NSCs). The remaining two mice were gravid females used for NSCs isolation.

Induction of ischemic brain stroke was performed through an application of an intraluminal filament (Doccol Corporation, Sharon, MA, USA, 6022910PK10Re) and by following modified Koizumi protocol, using 2% Isoflurane (Piramal Critical Care Limited, UK, 66794001725) in pure oxygen as inhalation anesthetic (Shahjouei et al., 2016). A modification in the protocol was made to reduce the number of suture knots. In total just two knots were placed below the bifurcation of the common carotid artery (CCA) into the external carotid artery (ECA) and internal carotid artery (ICA), a distal knot and a proximal knot. The distal knot was immediately tightened permanently, while the proximal knot was only loosely tightened to allow the intraluminal filament to pass through. An incision in CCA for inserting the filament was made between the distal and proximal knots. The intraluminal filament was inserted through the incision, and the proximal knot was tightened just enough to stop the bleeding. The occlusion lasted for 30 min. After this period, the filament was removed, and the proximal knot was tightened permanently. We found out that in this way, the animals have a higher survival rate, and MRI scans have shown that the strokes were still of proper size.

All experiments were approved by Internal Review Board of the Ethical Committee of the School of Medicine University of Zagreb (380-59-10106-17-100/27) and Ministry of Agriculture-Croatia (525-10/0255-17-6).

### 2.2 Isolation, cultivation, and transplantation of NSCs

NSCs were isolated from the telencephalic wall of 14.5 days old mice embryos (Alić et al., 2016). The cells were grown in suspension in a proliferation medium and transplanted after the 4th passage. The proliferation medium consisted of DMEM/F-12 (DMEM/F-12 (1:1) (1X) + GlutaMAXTM-I, Gibco by life Technologies, 31331-028) in which was added 2% B-27 (B-27®Supplement (50X), Gibco, 17504-044), 1% N-2 (N-2 Supplement (100X), Gibco, 17502-048), 1% Pen Strep (Penicillin Streptomycin, Gibco, 15070-063), 0.5% HEPES (4-(2-hydroxyethyl)-1-piperazineethanesulfonic acid, Gibco, 15630-056), 0.2% (10 μg/mL) FGFb (Recombinant Mouse Fibroblast Growth Factor-basic, Thermo Fischer Scientific, PMG0035) and 0.2% EGF (20 ng/mL) (Recombinant Mouse Epidermal Growth Factor, Thermo Fischer Scientific, PMG8041). The stereotaxic transplantation of 1 million cells was performed 24 h after stroke induction using Small Animal Stereotaxic Instrument Kopf 900LS (David Kopf Instruments, Tujunga, CA, USA) and Hamilton glass syringe (Hamilton Company, USA, #80100) under inhalation anesthesia targeting border between cortex and striatum, following coordinates determined according to stereotaxic atlas: AP +0.5, ML +2.3, DV -2.5 (Paxinos and Franklin, 2001).

### 2.3 Magnetic resonance imaging (MRI)

*In vivo* MR imaging was performed using 7 T BioSpec 70/20 USR MRI system (Bruker BioSpin, Ettlingen, Germany) with Paravison 6.0.1. software in a Tx/Rx configuration. The main scans included a high-resolution T2-weighted and a T2-map Multi-echo Spin-Echo sequence scan (Justić et al., 2022). Each animal was imaged 7 days before MCAO (BL), 24 h after MCAO (D1) and, for half of the animals from each group, 5 days after MCAO (D5).

### 2.4 Neurological status assessment

In order to quantify the level of impairment and rate of recovery following stroke, a scoring system based on the compilation of the commonly utilized ones was used (Schaar et al., 2010). A score of 0 means that an animal is completely healthy, while the maximum number of negative points was 39. Animals were assessed for appearance and behavior. A score of 0 was given for healthy, clean, and well-groomed fur, 1 for groomed fur with slightly ruffled hairs on the back, and 2 for neglected and dirty fur with ruffled hairs. For ears, a score of 0 was given if both ears were normally upright, 1 if one ear was drooping or pulled back, and 2 if both ears were drooping or pulled back. Eye assessment: 0 if both eyes were normal, 1 or 2 (L/R) if the eyes were slightly closed, and 3 or 4 (L/R) if the eyes were completely closed.

Animal posture was evaluated as follows: 0 points for normal posture, 1 for a slight arch on the back that straightens when walking, 2 for a larger arch that straightens when walking, 3 for a hump that is always present even when walking, and 4 points for the most severe hunch that interferes with walking.

A score of 0 was given for normal motility, 1 for slightly reduced exploratory behavior, 2 for moving limbs without proceeding, 3 for moving only to stimuli, 4 for unresponsiveness to stimuli with normal muscle tone, and 5 for severely reduced muscle tone and premortal signs. For gait disturbances, 0 was given for straight walking, 1 for walking toward the contralateral side, 2 for alternating circling and walking straight, 3 for alternating circling and walking toward the paretic side, 4 for circling and other gait disturbances, and 5 for constant circling toward the paretic side.

Forelimb flexion: 0 if both limbs were extended when held by the tail, 1 if just one limb was extended, and 2 if neither limb was extended. For the degree of body rotation when held by the tail, 0 was given if the animal flexed on both sides, 1 if flexed on just one side, and 2 if there was no flexion and the animal just hung.

For resistance against a lateral push, 0 was given for normal resistance, 1 for weak resistance, and 2 for no resistance. Forelimb placing on the ipsilesional side: 0 for normal placing, 1 for weak placing, and 2 for no placing. For the contralesional side: 0 for normal placing, 1 for weak placing, and 2 for no placing.

The acoustic startle reflex was tested with a clap; 0 was given if there was a response and 1 if there was no response. The whisker reflex was tested with a cotton swab on the left and right sides; 0 was given if there was a response and 1 point if there was no response on each side. The pinna reflex was tested with a cotton swab on the left and right ear; 0 was given if there was a response and 1 point if there was no response on each side. The proprioceptive reflex was tested by touching the legs with a cotton swab; 0 points were given if there was a reflex response of withdrawing the leg, and 1 point if there was no response on each side of the body.

### 2.5 Isolation and preparation of the brain tissue

To obtain the tissue for immunohistochemical analysis, the animals were anesthetized with an intraperitoneal injection of 2.5% tribromoethanol (2,2,2-Tribromoethanol, SIGMA, T48402-5G), then underwent transcardial perfusion with house made PBS 1X (8 g of NaCl, 1.44 g of Na_2_HPO_4_, 0.2 g of KCl, and 0.24 g of KH_2_PO_4_ in 1 L of dH_2_O. pH 7.4 adjusted with NaOH) and 4% formaldehyde (BIGNOST Ltd., HR, FNB4-1 L). The isolated brains were submerged in 4% formaldehyde for 24 h, washed in PBS and transferred to 30% sucrose in PBS. The tissue was imbedded in Tissue-Tek O.C.T. Compound, (Sakura Finetek, USA, 4583) and cryotome sectioning of 30 μm frontal sections. To obtain the tissue for qPCR animals were anesthetized with an intraperitoneal injection of 2.5% tribromoethanol, then underwent transcardial perfusion with PBS, and the brain hemispheres were snap frozen and dry pulverized in liquid nitrogen. Half of the animals from each group were sampled on the 2nd day, and the other half on the 5th day following stroke induction (Alić et al., 2016).

### 2.6 qPCR

The total RNA was isolated from pulverized tissue by using the commercial QIAshredder (Qiagen, Germantown, MD, USA, 79656) and RNeasy Mini Kit (Qiagen, 74104). The RNA concentration was quantified using NanoDrop ND1000 spectrophotometer (Thermo Fischer Scientific, USA) and based on measured values, it was determined that all samples for transcription would be standardized to contain 25 ng/μL of RNA. The conversion from RNA to cDNA was accomplished utilizing a high-capacity RNA-to-cDNA kit (Applied Biosystems, Bedford, MA, USA, 4387406). The qPCR was performed using TaqMan Gene Expression Assays for two genes of interest: Gasdermin D (*Gsdmd*) (Thermo Fisher Scientific, Mm00509962_g1) and mixed lineage kinase domain-like protein (*Mlkl*) (Thermo Fisher Scientific, Mm01244220_m1). As housekeeping genes Actin-β (*Actb*) (Thermo Fisher Scientific, Mm02619580_g1) and Hypoxanthine phosphoribosyltransferase 1 (*Hprt1*) (Thermo Fisher Scientific, Mm03024075_m1) were used. The relative quantification was performed using the ΔCT method (Hribljan et al., 2018).

### 2.7 Immunohistochemistry/immunofluorescence

The brain tissue samples were immunolabeled with specific primary antibodies diluted in PBS with 0.05% Tween 20 (Carl Roth GmbH, Karlsruhe, Germany, 9127.2) and goat serum (Thermo Fisher Scientific, 16210064) at 4°C overnight. Prior to immunostaining, the antigen retrieval was performed in citrate buffer, pH = 6.0 (Thermo Fischer Scientific, 00-4955-58). The primary antibodies were rinsed with PBS and the samples were immunolabeled with fluorescent secondary antibodies diluted in PBS. After rinsing the secondary antibodies with PBS, DAPI (Roche, Basel, Switzerland, 10236276001) was used as a nuclear counterstain. The samples were covered with coverslips with Dako Fluorescent Mounting Medium (Agilent, Santa Clara, CA, USA, S302380-2) and analysed using a confocal microscope OlympusFV3000 (Olympus, Tokyo, Japan) (Alić et al., 2016). Primary antibodies and dilutions used were: anti-GSDMD/1:300 (Abcam, ab219800), anti-phospho MLKL/1:400 (Cell Signaling. #37333), anti-GFAP/1:500 (Abcam, ab4674), anti-IBA1/1:100 (Invitrogen, MA5-27726), anti-MAP2/1:1000 (Abacam, ab5392), anti-NEUN/1:200 (Sigma-Aldrich, MAB377). Secondary antibodies and dilutions used were: Alexa Fluor 488/1:1000 (Life Technologies A11039), Alexa Fluor 488/1:1000 (Life Technologies A11001), Alexa Fluor 546/1:1000 (Life Technologies A11010), Alexa Fluor 647/1:500 (Abcam, ab150171), Alexa Fluor 647/1:500 (Abcam, ab150115).

### 2.8 Image analyses and statistics

For calculating the volumes of brain hemispheres, T2w MR images were used, while T2map MR images were used to quantify the stroke volume. All the images were processed by manual delineation of hemispheres using the ImageJ software. In total 76 brains were scanned, with 20 of them belonging to the control and 56 to the stroke-affected group. Since repeated measures ANOVA cannot handle missing values that occurred due to decreased number of mice in later time points, some of the obtained raw data has been analysed by fitting a mixed model with a Geisser–Greenhouse correction. While MRI and qPCR data analysis used Tukey Kramer multiple comparisons *post-hoc* test, the *post-hoc* analysis of the neuroscoring data was performed with Šidák’s multiple comparisons test to characterize the differences between the groups. This analysis was implemented in GraphPad Prism version 9.3.1.

For performing the analyses of signal intensity, surface covering the visual field and colocalization of two or more signals, the CellProfiler software was used. For this purpose, we used 3 different brains for each gene and scanned 15 images for each brain and for each combination of markers. After manually preparing a pipeline with an acceptable signal-noise ratio, all the images were analysed with an automatic protocol as a single batch. After obtaining the raw data for percentage of slide coverage and mean intensity as well as colocalization through image analysis the parameters were statistically analysed using a two-way ANOVA with Tukey Kramer multiple comparisons *post-hoc* test. The significance levels are as follows: *p ≤ 0.05, **p ≤ 0.01, ***p ≤ 0.001 and ****p ≤ 0.0001.

## 3 Results

### 3.1 Transplanted NSCs improved recovery of mice affected by stroke

**Figure 1:**
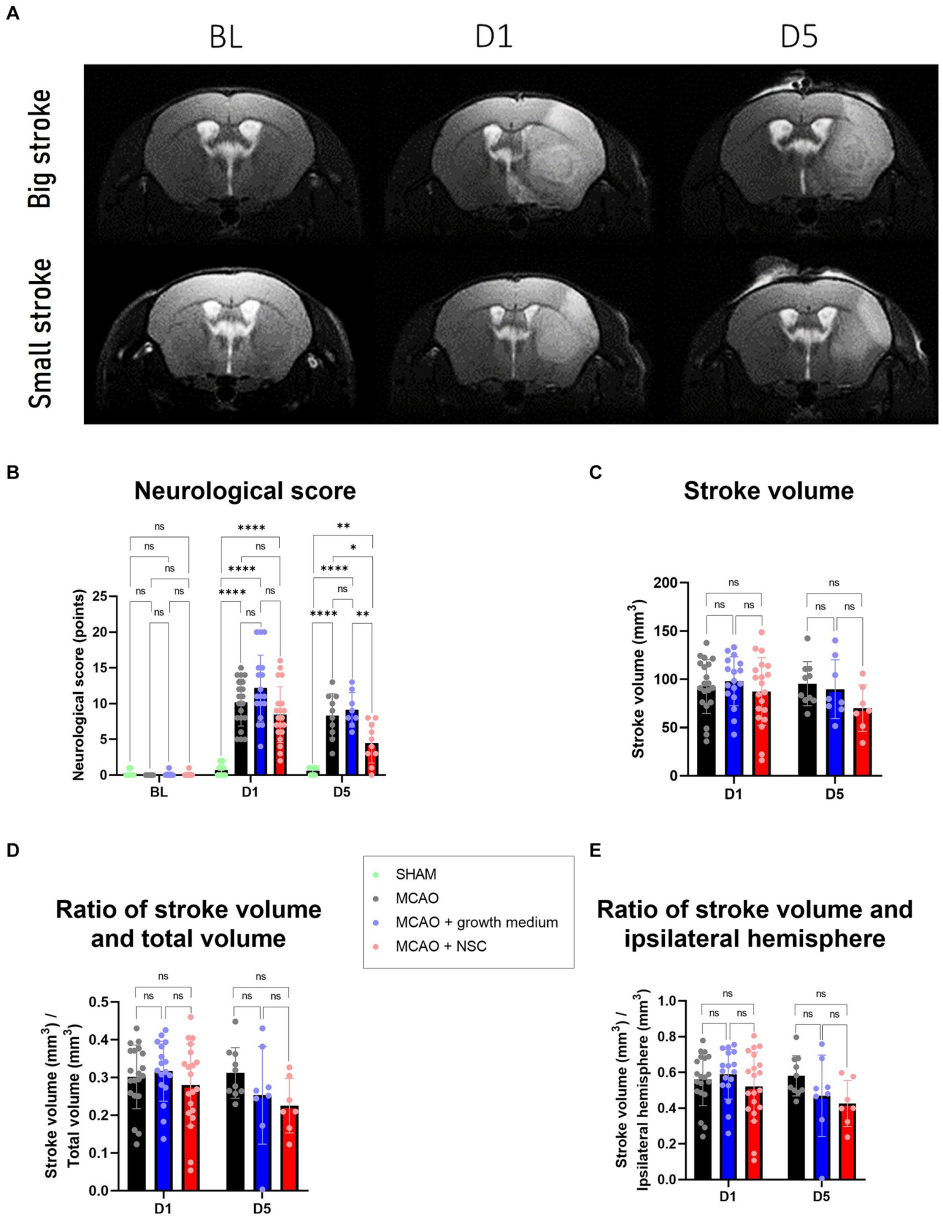
**(A)** MRI scans allowed for measurement of stroke volume on days 1 and 5, as compared to the baseline (BL) image. Upper row represents a big stroke, lower row represents small stroke. **(B)** Neurological score, assessed as described in paragraph 2.4., revealed worsening of the health condition after onset of stroke, which was significantly improved only in the group treated by NSCs (group D), as visible after 5 days. **(C)** Stroke volume, measured by manual delineation in ImageJ from MRI images, was slightly reduced in animals treated by transplantation of medium and more reduced in animals treated by NSCs. D and E: The ratios of stroke vs. the total and ipsilateral brain volume, all measured by manual delineation of MRI images in ImageJ, revealed tendency of improvement in the animals treated by NSCs. The significance levels are as follows: *p ≤ 0.05, **p ≤ 0.01, ***p ≤ 0.001 and ****p ≤ 0.0001.

Following ischemic stroke, as visualized by magnetic resonance imaging (MRI) (Figure 1A), the analysis of obtained neurological scores, in all the operated groups on day 1 (D1), revealed a significant worsening in the animal’s status compared to sham operated animals (Figure 1B). The comparison of scores between groups on D5 (4 days after NSCs transplantation, cell medium or no treatment) clearly revealed that animals which received NSCs transplantation achieved significantly improved levels of recovery (Group D), when compared with both animals which received basal NSCs medium (Group C) and stroke affected animals (Group B) (Figure 1B).

### 3.2 Transplanted NSCs reduced the stroke volume in mice

On the day 1, the average stroke volume was approximately 100 mm^3^ across all groups with no significant differences. Interestingly, by day 5, variations emerged: the untreated group maintained a volume of around 100 mm^3^, the NSCs medium group (Group C) decreased to an average of 91 mm^3^, and the NSCs transplanted group (Group D) showed a volume of 80 mm^3^ (Figure 1C). Despite noticeable reductions, these differences were not statistically significant (*p* values between groups B-C, B-D, and C-D for D1 and D5 are listed respectively: 0.8582, 0.8372, 0.5349, 0.8918, 0.1381, 0.3211). Exploring additional parameters, we compared stroke volume to the whole brain and ipsilateral hemisphere volumes revealing some distinctions in the NSCs-injected group, although not significant we showed the same trend (*p* values for ratio of stroke volume and total brain volume between groups B-C, B-D, and C-D for D1 and D5 are listed respectively: 0.8693, 0.7395, 0.4488, 0.3844, 0.1630, 0.8369 and *p* values for stroke volume and ipsilateral hemisphere volume between groups B-C, B-D, and C-D for D1 and D5 are listed respectively: 0.8441, 0.7292, 0.4104, 0.3314, 0.1517, 0.8660) (Figures 1D,E).

### 3.3 Transplanted NSCs upregulated the expression of *Gsdmd* while lowering GSDMD signal intensity

**Figure 2:**
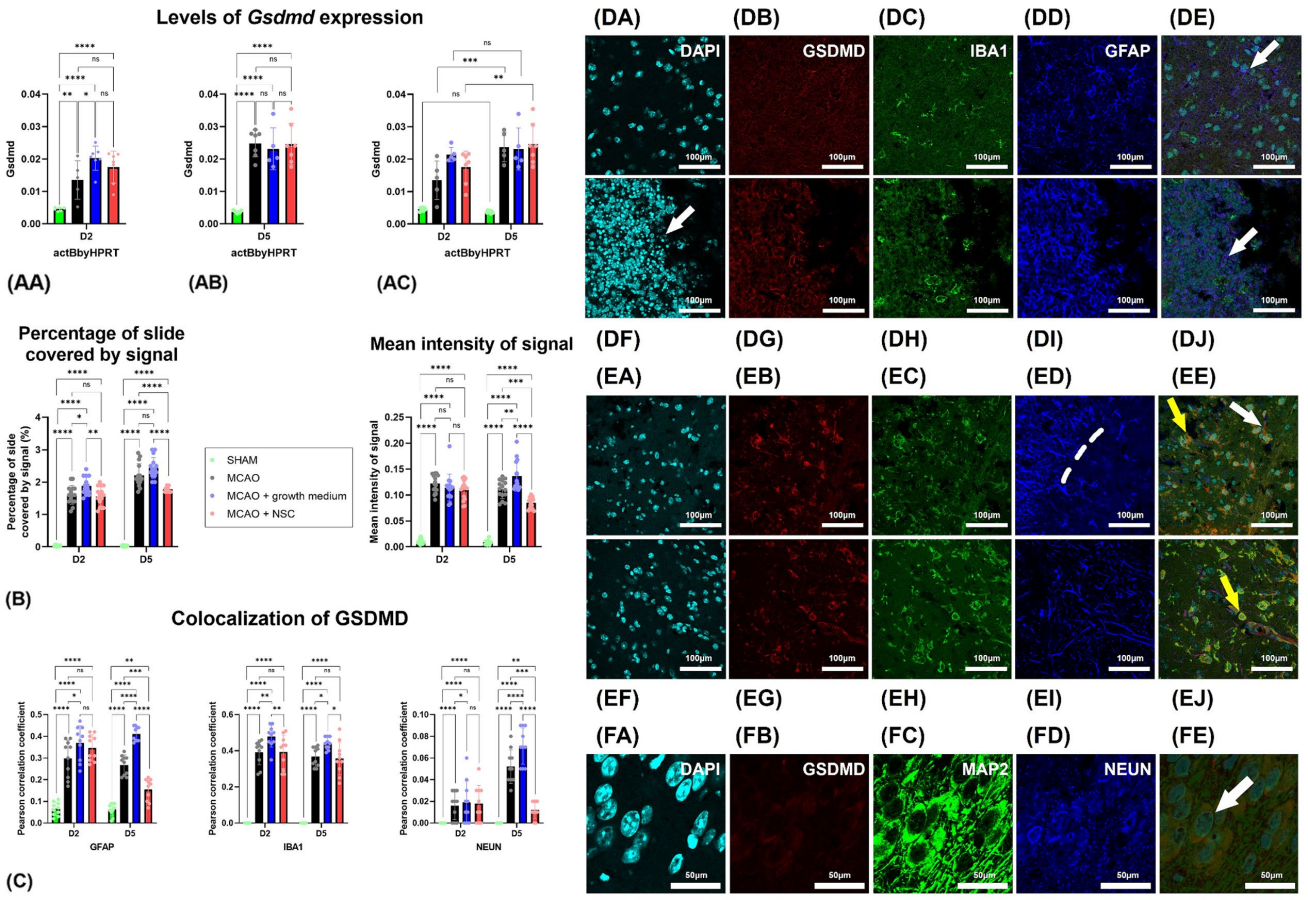
Stroke increased *Gsdmd* expression quantified by qPCR in all groups, with no treatment reducing it after 5 days. **(AA)** Two days post-stroke, the highest increase was in the NSCs medium-treated group (Group C), followed by the NSCs-treated group (Group D). **(AB)** Five days post-stroke, expression levels were higher overall but more uniform across groups. **(AC)** Comparing same groups on different timepoints showed that *Gsdmd* expression of the cell-treated (Group D) and non-treated groups (Group B) showed significant upregulation, while the medium-treated group (Group C) showed upregulation without statistical significance. **(B)** Intensity of the total *Gsdmd* signal and percentage of slide coverage, measured on immunofluorescence images in CellProfiler, increased after stroke and was significantly moderated by NSCs 4 days after transplantation. **(C)** Colocalization analyses, measured on immunofluorescence images in CellProfiler, revealed that after stroke GSDMD colocalizes dominantly with GFAP and IBA1 positive cells. Four days after transplantation of NSCs, colocalization of GSDMD and GFAP is strongly reduced. **(DA–DE)** Immunohistochemical analyses on day 2 revealed colocalization of GSDMD with both astrocytes (white arrow) and microglia (yellow arrow). Moreover, GSDMD-positive cells were prominently present in penumbra region near the hypoxic core. There was no visible difference in animals which received NSCs, 1 day after transplantation **(DF–DJ)**. **(EA–EE)** On day 5 colocalization with both astrocytes (white arrow) and microglia (yellow arrow) was found. Transplantation of NSCs significantly reduced presence of GSDMD in astrocytes 5 days after transplantation **(EF–EJ)**. **(FA–FE)** Colocalization of GSDMD and markers of neurons was rather rare finding. Scale bar on images **(DA–EJ)** is 100 μm, while on **(FA–FE)** is 50 μm. The significance levels are as follows: *p ≤ 0.05, **p ≤ 0.01, ***p ≤ 0.001 and ****p ≤ 0.0001.

Stroke enhanced the expression of *Gsdmd* on RNA level in all groups, and none of the treatments reduced it even after 5 days. Two days post-stroke and 1 day post-NSCs injection, *Gsdmd* expression showed the greatest increase in the NSCs medium-treated group (Group C), followed by the NSCs-treated group (Group D), while untreated animals showed lower levels. Gene expression analysis 5 days post-stroke (4 days post-transplantation) revealed higher overall values with more uniformity among groups (Figures 2AA,AB). However, there was no statistically significant difference between groups on both days. Comparing same groups in different time points showed statistically significant upregulation for cell treated group and non-treatment group, while results in medium-treated group still showed upregulation but with no statistical differences (Figure 2AC).

Analysis of GSDMD immunohistochemistry signal intensity and coverage revealed increased cell presence 2 days post-stroke. No significant difference appeared 1 day post-cell transplantation. However, by day 5 post-stroke and 4 days post-transplantation, NSCs-transplanted animals (Group D) exhibited statistically significantly reduced slide coverage and overall signal intensity (Figure 2B).

Two days post-stroke with no treatment (Group B), GSDMD-positive cells were prominently present in penumbra region near the hypoxic core, with a majority co-expressing IBA1 or GFAP, indicating expression in both microglia and astrocytes (Figures 2DA–DE). In NSCs transplanted animals (Group D) (Figures 2DF–DJ), 1 day post-procedure, IBA1 and GFAP positive cells persisted (Figure 2, compare B-untreated vs. G-cell treated). A similar pattern was observed 5 days post-stroke (Figures 2EA–EE), with clear GSDMD colocalization with IBA1 and GFAP in brain regions near the hypoxic core. Notably, in transplanted animals (Figures 2EF–EJ), the colocalization of GFAP and GSDMD was less frequent than in untreated animals. Analysis of neuronal markers (MAP2 and NEUN) revealed rare GSDMD-positive neurons in both treated and untreated groups at both time points (Figure 2FA–FE). Contralateral hemisphere analysis showed fewer and less intense GSDMD-positive cells compared to the stroke-affected hemisphere (data not shown).

Two days post-stroke, GSDMD predominantly colocalized with IBA1, slightly less with GFAP, and almost none with NEUN. NSCs transplantation showed no significant impact on this pattern. However, by day five post-stroke, in the untreated group, GSDMD was mainly present with IBA1, slightly less in GFAP, and rarely in NEUN positive cells. In contrast, NSCs-transplanted animals exhibited reduced GSDMD presence in GFAP, with no impact on IBA1 colocalization. Furthermore, NSCs transplantation significantly reduced GSDMD presence in neurons, despite their scarcity both two- and five-days post-stroke (Figure 2C).

### 3.4 Transplanted NSCs downregulated the expression of *Mlkl*, decreased pMLKL in neurons and increased its distribution in microglia

**Figure 3:**
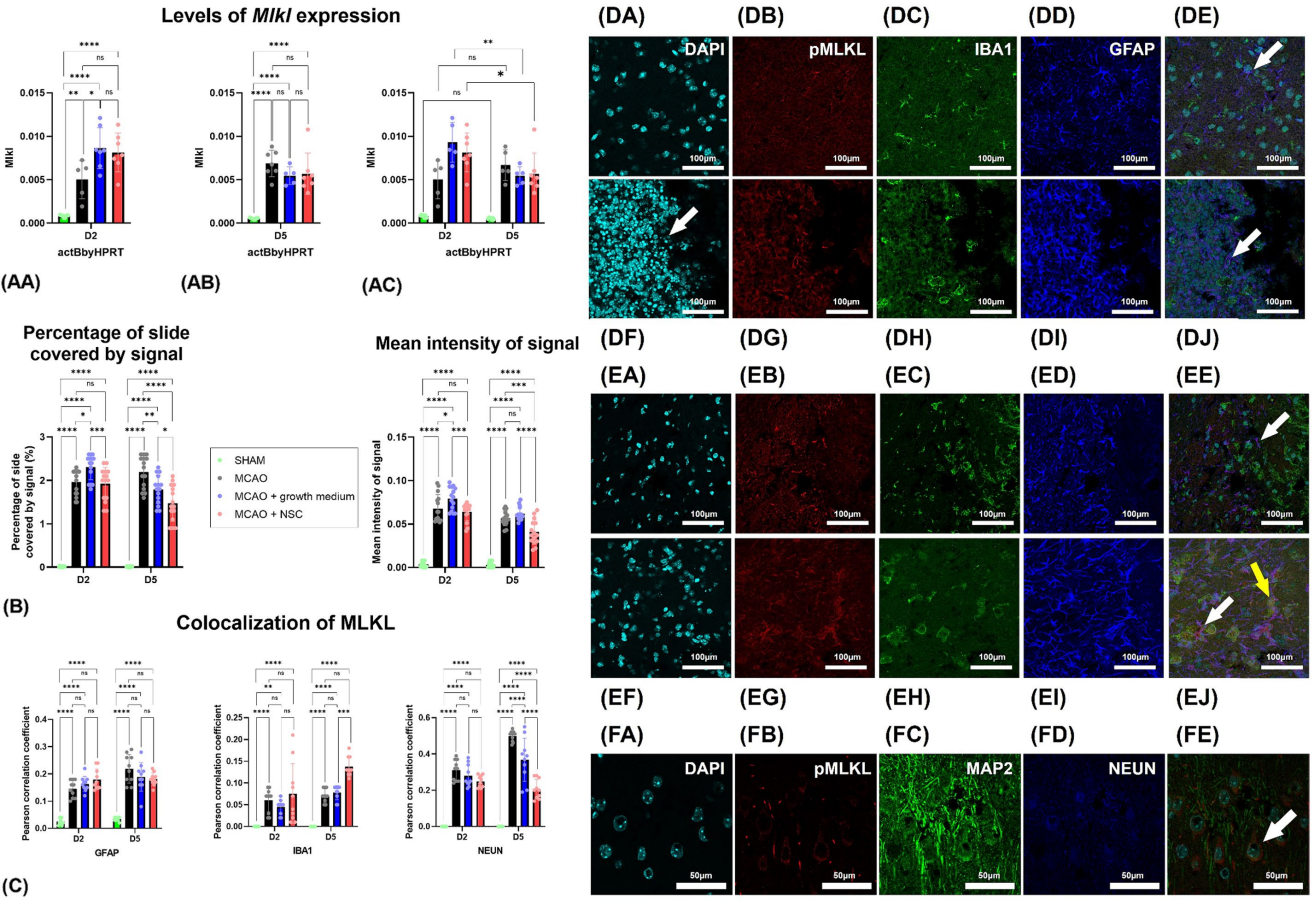
**(AA)** Two days post-stroke, *Mlkl* expression quantified by qPCR increased in both medium-treated and NSCs-transplanted groups, while untreated animals showed lower levels. **(AB)** Five days post-stroke, untreated animals had increased *Mlkl*, while medium-treated and NSCs-treated groups showed lower expression. **(AC)** Comparing gene expression at different timepoints showed significant downregulation of *Mlkl* in medium-treated and NSCs-treated groups, while the non-treated group had a slight, non-significant upregulation. **(B)** Intensity of the total pMLKL signal and percentage of slide coverage, measured on immunofluorescence images in CellProfiler, increased after stroke and was significantly moderated by NSC 4 days after transplantation. **(C)** Colocalization analyses, measured on immunofluorescence images in CellProfiler, revealed that after stroke pMLKL colocalizes dominantly with GFAP and NEUN positive cells. Four days after transplantation of NSCs, colocalization of pMLKL and NEUN was strongly reduced, while colocalization of pMLKL and IBA1 was increased. **(DA–DE)** Immunohistochemical analyses on day 2 revealed abundant pMLKL-positive cells near the hypoxic core and colocalization of pMLKL with astrocytes (white arrow). There was no visible difference in animals which received NSC, 1 day after transplantation **(DF–DJ)**. **(EA–EE)** On day 5 colocalization with astrocytes (white arrow) was found. Transplantation of NSC significantly reduced presence of pMLKL in astrocytes, but increased presence of pMLKL in microglia (yellow arrow) 5 days after transplantation **(EF–EJ)**. **(FA–FE)** Colocalization of pMLKL and neuronal markers. Scale bar on images **(DA–EJ)** is 100 μm, while on **(FA–FE)** is 50 μm. The significance levels are as follows: *p ≤ 0.05, **p ≤ 0.01, ***p ≤ 0.001 and ****p ≤ 0.0001.

Two days post-stroke and 1 day post-stem cell treatment, *Mlkl* expression increased in both medium-treated and NSCs transplanted groups, while untreated animals showed lower levels of expression (Figure 3AA). Gene expression analysis 5 days post-stroke (4 days post-transplantation) indicated an increase in the untreated group, with lower expression in the medium-treated and NSCs-treated groups (Figure 3AB). Comparing gene expression within the same groups at different timepoints revealed statistically significant downregulation in the medium-treated and NSCs-treated groups, while the non-treated group exhibited a small upregulation, though not statistically significant (Figure 3AC).

Quantification of pMLKL signal intensity through immunohistochemistry showed that 2 days post-stroke, there was a dramatic increase in pMLKL signal intensity and coverage in cells. One day post-NSCs transplantation and 2 days post-stroke showed no significant difference between treated and untreated groups. However, by day 5 post-stroke and 4 days post-transplantation, animals receiving stem cells exhibited significantly lower slide coverage and overall intensity of the pMLKL signal (Figure 3B).

Two days post-stroke, immunohistochemical analysis revealed abundant pMLKL-positive cells near the hypoxic core, predominantly neurons and astrocytes, with rare microglia positive for pMLKL (Figures 3DA–FE). Comparing this to animals receiving cell transplantation showed no obvious difference 1 day post-transplantation (Figures 3DF–DJ). By day 5 post-stroke, pMLKL-positive microglia became more common (Figures 3EA–EJ). Analysis of neuron markers (MAP2 and NEUN) showed widespread pMLKL presence in neurons, a common finding in both treated and untreated animals at both time points (Figures 3FA–FE).

Two days post-stroke, pMLKL primarily colocalized with neurons, with rare colocalization in astrocytes and microglia. By day 5 post-stroke, colocalization with neuronal markers increased, while astrocyte and microglia presence remained unaffected. Notably, NSC transplantation significantly influenced these patterns: astrocyte colocalization remained unaffected, but NSCs increased pMLKL in microglia and decreased it in neurons (Figure 3C).

## 4 Discussion

Decades of testing stem cell therapy in animal stroke models demonstrate measurable benefits. A meta-analysis of 37 preclinical trials with NSCs reveals consistent improvements in motoric capabilities, with less consistent results in neurological scores and lesion volume (Chen et al., 2016). These promising findings have propelled the therapy into advanced clinical trials, primarily addressing its potential to accelerate human recovery, after early safety assessments (Jaillard et al., 2020; Muir et al., 2020; Lee et al., 2022). However, failure of many clinical trials suggested a need for further improvements of pre-clinical phases. Our group contributed to those endeavors by improving *in vitro* studies, both from methodological points of view (Petrović et al., 2023; Radoszkiewicz et al., 2023), and by focusing on specific molecular elements of events followed by hypoxic injury (Jagečić et al., 2023; Stančin et al., 2023).

In further improvement of understanding the therapeutic mechanisms, the pivotal role of growth factors, released by NSCs, stands out as a central event, contributing to reduced inflammation (Bacigaluppi et al., 2009; Huang et al., 2014; Hamblin et al., 2022). These growth factors, known for their neuroprotective and regenerative effects (Leker et al., 2009), were key players in the observed improvements, with limited studies emphasizing a reduction in apoptosis as a foundational aspect (Shen et al., 2010).

Our focus on pyroptosis and necroptosis stems from the robust neuroinflammatory response triggered after stroke, leading to cytokine release and danger-associated molecules. The activated cells were predominantly in the penumbra rather than the necrotic core. Thus, our morphological analyses concentrated on this region, revealing a significant presence of cells expressing markers for pyroptosis and necroptosis.

After confirming that *Gsdmd* is expressed at very low levels under normal conditions, but strongly upregulated after onset of stroke, we found that a great majority of GSDMD positive cells were microglia and astrocytes but not neurons, and similar results were shown by other authors (Wang et al., 2020). Transplantation of NSCs yielded interesting effects 4 days after the transplantation (5 days after onset of stroke), which were visible in the general increase of expression of *Gsdmd*, but on the other hand dominant overall reduction of this marker’s presence, especially in astrocytes, suggesting that transplanted NSCs affect GSDMD activity. Our results can also be compared to some studies in which the authors reported that intravascular transplantation of either olfactory mucosa stem cells or exosomes into a rat stroke model reduces pyroptosis (Liu et al., 2021; Zhuo et al., 2021). However, in both studies, the reduction of pyroptosis was measured on a protein level of a whole brain and analyses of specific cell populations were not performed. We showed that despite gene upregulation in treated groups, transplanted NSCs can reduce activity of GSDMD, predominantly in astrocytes, in stroke affected mouse brain which improved animal recovery. Other studies have also proven that reducing the activity of GSDMD in cells can have significant impact on recovery after ischemic brain stroke, either by pharmacological intervention or by using knockout animals (Wang et al., 2020; Hu et al., 2023). One study proved that stem cells conditioned medium can reduce pyroptosis by inhibiting the NLRP3/caspase-1/interleukin-1β pathway (Liu et al., 2024). It is plausible to hypothesize that at least a part of positive effects can be contributed to the secretion of molecules which decrease levels of inflammatory factors such as caspase-1, NLRP3, GSDMD, IL-1β, and IL-18 (Li et al., 2019; Liang et al., 2021).

The study we conducted revealed dynamic changes in *Mlkl* expression and pMLKL distribution post-stroke, along with findings from other authors (Li et al., 2020). Expression of *Mlkl* went down after cell treatment indicating reduction of necroptosis. Kong et al. reported reduction of necroptosis by addition of MSCs reducing the levels of RIPK1 and RIPK3 in an *in vitro* model of ischemia (Kong et al., 2016). As such, our study is demonstrating the reduction of necroptosis using NSCs on *in vivo* system, rather than on *in vitro* system. Moreover, and different from reports based on RIPKs, we performed our analyses by quantifying the distribution of pMLKL, which is considered the ultimate proof of active necroptosis (Galluzzi et al., 2014). Immunohistochemistry also showed a surge in pMLKL intensity, especially in neurons and astrocytes where NSCs-treated animals displayed significantly reduced intensity by day 5. Colocalization studies demonstrated that initially, pMLKL primarily colocalized with neurons, which was shown by others as well (Han et al., 2019). NSCs treatment influenced this pattern, increasing pMLKL in microglia and decreasing it in neurons. The acknowledged role of inflammation in triggering necroptosis involves key inflammatory markers such as TNF-α, IL-1β, IL-1α, and IL-6 (Zhang et al., 2016; Yang et al., 2017), with NSCs demonstrating the potential to mitigate proinflammatory cytokines (Huang et al., 2014). Since the reduction of necroptosis by its pharmacological inhibitor has also been shown as beneficial in brain ischemia (Northington et al., 2011; Mitroshina et al., 2023), our findings of reduced necroptosis in neurons following NSCs transplantation are in accordance with aforementioned literature and our reports of reduction in stroke volume and accelerated recovery of mice.

In conclusion, we showed the measurable benefits of NSCs therapy targeting pyroptosis and necroptosis in stroke recovery. NSCs, known for releasing growth factors with neuroprotective effects, show promise in reducing inflammation. Our study delves into pyroptosis and necroptosis post-stroke following NSCs transplantation, revealing *Gsdmd* upregulation, but reducing GSDMD signal intensity in microglia and astrocytes. NSCs transplantation influences necroptosis, leading to its reduction, as indicated by pMLKL intensity reduction in neurons and astrocytes. These findings align with the acknowledged role of inflammation in necroptosis and demonstrate NSCs’ potential to mitigate proinflammatory cytokines. The observed reduction in stroke volume and accelerated recovery further underscores the therapeutic potential of NSCs in stroke treatment.

## Data availability statement

Publicly available datasets were analyzed in this study. This data can be found here: https://doi.org/10.5281/zenodo.13136667.

## Ethics statement

The animal studies were approved by Internal Review Board of the Ethical Committee of the School of Medicine University of Zagreb. The studies were conducted in accordance with the local legislation and institutional requirements. Written informed consent was obtained from the owners for the participation of their animals in this study.

## Author contributions

DL: Data curation, Formal analysis, Investigation, Methodology, Resources, Software, Visualization, Writing – original draft, Writing – review & editing. IA: Investigation, Software, Supervision, Validation, Writing – original draft, Writing – review & editing. IŠ: Methodology, Software, Validation, Writing – original draft, Writing – review & editing. DM: Conceptualization, Funding acquisition, Investigation, Methodology, Resources, Supervision, Validation, Writing – original draft, Writing – review & editing.
